# Palatal development of preterm and low birthweight infants compared to term infants – What do we know? Part 3: Discussion and Conclusion

**DOI:** 10.1186/1746-160X-1-10

**Published:** 2005-11-02

**Authors:** Ariane Hohoff, Heike Rabe, Ulrike Ehmer, Erik Harms

**Affiliations:** 1 Poliklinik für Kieferorthopädie, Universitätsklinikum, Westfälische Wilhelms-Universität, Münster, Germany; 2 Department of Neonatology, Brighton & Sussex University Hospitals, UK; 3 Klinik für Kinderheilkunde, Division of Neonatology, Universitätsklinikum, Westfälische Wilhelms-Universität, Münster, Germany

## Abstract

**Background:**

It has been hypothesized that prematurity and adjunctive neonatal care is 'a priori' a risk for disturbances of palatal and orofacial development which increases the need for later orthodontic or orthognathic treatment. As results on late consequences of prematurity are consistently contradictory, the necessity exists for a fundamental analysis of existing methodologies, confounding factors, and outcomes of studies on palatal development in preterm and low birthweight infants.

**Method:**

A search of the literature was conducted based on Cochrane search strategies including sources in English, German, and French. Original data were recalculated from studies which primarily dealt with both preterm and term infants. The extracted data, especially those from non-English paper sources, were provided unfiltered in tables for comparison (Parts 1 and 2).

**Results:**

Morphology assessment of the infant palate is subject to non-standardized visual and metrical measurements. Most methodologies are inadequate for measuring a three-dimensional shape. Several confounding factors were identified as causes contributing to disturbances of palatal and orofacial development.

**Conclusion:**

Taking into account the abovementioned shortcomings, the following conclusions may be drawn for practitioners and prospective investigators of clinical studies. 1) The lack of uniformity in the anatomical nomenclature of the infant's palate underlines the need for a uniform definition. 2) Metrically, non-intubated preterm infants do not exhibit different palatal width or height compared to matched term infants up to the corrected age of three months. Beyond that age, no data on the subject are currently available. 3) Oral intubation does not invariably alter palatal morphology of preterm and low birthweight infants. 4) The findings on palatal grooving, height, and asymmetry as a consequence of orotracheal intubation up to the age of 11 years are inconsistent. 5) Metrically, the palates of orally intubated infants remain narrower posteriorly, beginning at the second deciduous molar, until the age of 11 years. Beyond that age, no data on the subject are currently available. 6) There is a definite need for further, especially metrical, longitudinal and controlled trials on palatal morphology of preterm and low birthweight infants with reliable measuring techniques. 7) None of the raised confounding factors for developmental disturbances may be excluded until evident results are presented. Thus, early orthodontic and logopedic control of formerly premature infants is recommended up to the late mixed dentition stage.

## Background

The research on palatal development dates back to the beginning of the last century. Unfortunately, the results of many studies are conflicting in some respects and may be difficult to interpret. Compared to the significant improvements in the survival rate of preterm infants, the knowledge on late consequences of orofacial development in these small patients is still unsatisfactory. A recently published systematic review concluded that further well-designed studies are needed [1]. Therefore, a fundamental analysis of existing methodologies, confounding factors, and outcomes seems motivated.

Traditional reviews are characterized by less stringent inclusion criteria than systematic reviews. Thus, studies from the pre-'evidence based medicine' (EBM) era were included. This has revealed important information about the research on development of the term infant's palate. It could be shown (Part 1) that the newborn's palate can vary considerably and is subject to various influences. Therefore, palatal growth may occur undetected or appear excessive or inadequate.

The general methodologies used for morphometry are comparable between studies from the pre-EBM and EBM eras, whereas the quality of study designs improves over the years. The assessment of palatal alterations is only as accurate as the measurements that are taken from the palate and therefore independent of the general study type. Two-dimensional measurements – however reliable – are of limited value for the description of three-dimensional shape changes, and this measurement technique may contribute conflicting results.

The present part of the review discusses the different results of Parts 1 and 2. Conclusions were drawn based on clinical relevance and the quality of included studies.

## Discussion

### Clinical relevance of visual inspection of the newborn palate

Palatal height is often employed as a diagnostic criterion of craniofacial syndromes [2], as is an abnormal number of frenula [3]. Thickening of the alveolar ridges may be congenital or acquired, for example following prolonged anticonvulsant therapy [3]. There is the risk that thickened (lateral) palatine and alveolar ridges producing narrowness of the palate may give a false impression of increased palatal height [3,4]. The latter is a less common anomaly, but may be a manifestation of a number of syndromes [2,5]. The distinction is important clinically, since prominent lateral palatine ridges most commonly imply a long-term deficit of neuromuscular function and thus may be an important diagnostic clue to alterations dating back to early prenatal development. Prominent lateral palatine ridges are, for example, a characteristic feature of Smith-Lemli-Opitz syndrome [6]. They are also a feature in Apert's syndrome [7,8] and in disorders in which the tongue is small, displaced or immobilized, including a narrow palate, as well as in mucopolysaccharidosis and other storage diseases with an abnormal accumulation of various metabolic substances within the connective tissue [4].

### Clinical relevance of a metric description of the palatal configuration

'*Growth is the essence of the developing system. ... Growth of different parts of the body follows a predictable schedule during normal development and maturation. This timetable of development is influenced and controlled by many genetic and environmental factors. Any disturbance in the normal schedule of development and growth may lead to disproportion of physical features. These imbalances may be transient and can sometimes be compensated for by later catch up growth*' [3].

In order to establish whether a palate is normal, it is necessary to have reliable information on the extent of variation of the normal gum pad [9]. '*There is a definite need for standards of oral-facial dimensions in children within ... this age range*' (6 weeks to 36 months of age) [10]. The information would be extremely valuable for the health care professional treating posttrauma patients or patients with craniofacial anomalies [9,10]. Manufacturers also would benefit from metrical information [11] for example for designing appropriate pacifiers. Data for height and weight are available up to 36 months of age, but there is lack of information on oral dimensions [10].

Palatal height is often employed as a diagnostic criterion of craniofacial syndromes [5]. It was suggested, as a rough guide, that '*when maximum palatal height is greater than twice the height of the teeth, it should be considered abnormally high*' [3].

### Consequences of intubation on the maxillofacial region

PT and LBW infants often require short or long-term neonatal intubation for resuscitation and to relieve respiratory distress [12].

Complications resulting from intubation, however it is performed, are always to be expected: in cases of nasal intubation, potential problems are nasal deformation [13-16] and subsequent choanal stenosis. Airway obstruction, possible hypoxia [175] or respiratory problems may occur in cases of nasogastric tubes [17]. Neonates and small infants are nasal breathers due to immature coordination of respiratory and oral function [18]; only 6% of PT infants (gestational age 31 – 32 weeks) are able to breathe orally [19]. Because PT neonates are nose breathers orotracheal intubation is often preferred to nasal intubation [20], but also reported to be associated with a higher rate of unplanned extubation [21].

As the palatal bones of fetuses are spongy and connective tissue interspersed at the midline forms a weakened palatal configuration, oral defects can easily result from the trauma of oral intubation [22]. This may result in the inability of the tongue to meet the palate correctly [22] and may give rise to considerable functional impairment [20] like sucking problems and impaired middle ear function [15] or articulation disturbances [15,23], e.g. in the form of a significantly higher incidence of fair or poor speech intelligibility in contrast to non-orally intubated infants [22,24].

The following dental complications are described as potential consequences of oral intubation and can be either caused by lack of oxygen, by the larynoscope blade [25] or by the tube itself [26,27]: enamel hypoplasia in 18 – 70% of preterm neonates [24,25,28,29], severe disruption of the developing enamel organ and deviation of the crown/root angulation [30], dilaceration of primary teeth [28,31], retarded eruption of primary teeth [26,32,33], impaired amelogenesis [24,27,34-36], effects on the position of the central incisors [15].

Palatal complications reported in connection with oral intubation are erosion and indentation of the alveolar ridge [37], notching [30,32,38], a high [12,28,39], and narrow [39,40] palatal shape, asymmetry of the palate [12,22] and cleft palate [41]. It was recommend not to use the term clefting [28], since no oral nasal communication has been demonstrated [41,42].

Alveolar grooving [28], and 'palatal grooving' [12,24,28,30,38,39,39,41-46] have also been described, never occuring in combination [28]. The majority of articles dealing with the phenomenon fail to give a definition of palatal grooving. However, there are three exceptions:

1. Two authors defined a palatal groove as follows: '*Narrow channel of variable depth located near the midline of the palate as identified by visual inspection of the maxillary cast*' [39] (Comment: Consider the variability of the term 'narrow').

2. Two other authors, performing intraoral measurement with a micrometer '*from its floor to the surface of the palate at the midpoint of the hard palate*', selected a palatal groove of ≥0.5 cm arbitrarily as significant [15] (Comment: Consider how difficult it is to make precise intraoral measurements in a tiny infant).

3. A further group stated: '*By definition, a palatal groove is an architechtural deformity of the palate caused by external pressure from the orotracheal tube*' [47].

There are various hypotheses on the cause of grooving:

1. It is an oral manifestation of head flattening commonly seen in very premature infants [48]. The same compressive interplay of forces that contribute to craniofacial narrowing is transferred from the zygomatic structures through the lateral aspects of the hard palate and causes the palatal grooving [49].

2. The deformation results from continuous pressure of the endotracheal tube against the median palatine suture [28,38,39,41,50]. This might be aggravated by the direction of pressure applied to the front of the tube in order to hold it in its desired position [47] and also by sucking [9] and result in a pressure necrosis [37].

3. The groove is caused by constriction of the palate adjacent to the tube [47]. This broadening of the alveolar ridges creates the false impression that the palate has been eroded as a groove; in fact, the palate is intact but partially obscured [50-52]. This finding is confirmed metrically [32]: '*Palatal grooving did not always correspond with relative palatal depth, but did usually occur in intubated infants. We therefore consider that palatal grooving is not caused by the direct pressure of the orotracheal tube. It is more likely that it is due to overgrowth of the lateral palatine ridges*'. In their reply to a letter from Ginther [50], Molteni and Bumstead [53] agreed that the term 'palatal groove' might be misleading. '*Groove does not imply a palatal defect or cleft but rather a transient mechanical obstruction to normal ingrowth of the lateral palatine ridges.*'

4. Several authors [4,28,50-52] regard an impeded tongue function as the cause of the palatal deformation. Grooving was observed even when the tube did not have a midline location, as there was also an absence of tongue thrust against the palatal shelves, which allowed the shelves to grow together [52].

Unusually prominent lateral palatine ridges have been regarded as a nonspecific feature of a variety of disorders in which there is either a neuromotor dysfunction or a malformation which prevents tongue thrust into the palatal vault, suggesting that a long-standing deficit of tongue thrust is the common pathogenetic mechanism [4,50]. This is frequently associated with reports of a poor sucking reflex in early childhood. In most of these conditions the ridges ultimately appear to attain a normal adult configuration [4,50]. These authors believe, however, that truly narrow, highly arched palates are a very infrequent occurrence and are confused with primarily structural aberrations of the maxilla or the palate, or with prominent lateral palatine ridges.

At age 3–5 a characteristic high palatal vault on the dental casts of formerly intubated children was still observed, and 21% of the intubated infants with high palatal vaults also had palatal grooves; nearly 1/4 of the children suffered from crossbites; neither birthweight nor intubation was related to palatal symmetry [24]. No data was given in the abstract concerning the method nor if preterm or term children were examined.

### Quality of studies

Bias, i.e. wrong selection of included and excluded studies could have come over the presented review for several reasons.

Firstly the authors were already strongly involved in the matter and thus not blinded to its subject. Secondly, in only four of the 'metrical control studies' did the authors state explicitly that fullterm infants had been investigated [9,54-56]. In additional two studies, data for term infants could be extracted by the authors of the review because all single figured concerning weight or maturity were given by the authors of these dissertations [57,58]. In most of the metrical studies with term infants, the reliability of the method was not given. We have to comment that the data included in the study are the best evidence we have for the moment concerning a 'control group' of term infants.

Thirdly, non metrical studies and studies without dental casts should be interpreted with caution due to several shortcomings: they suffered from a lack of definition or a non-uniform definition of the term '*palatal grooving*', from low case numbers in some studies, from the difficulty of intraoral assessments in very small babies and from subjective assessment of palatal shape. In some studies, the intra-examiner reliabilty was not given [43,44] or statistically significant inter-examiner differences existed [43], whereas in one paper the subjective assessment of relative palatal height turned out to be fairly reliable (approximately 80% inter-and intraexaminer agreement) [59].

Visual descriptions alone cannot always give rise to valid decisions on whether the alterations described in the palatal configuration are in fact palatal grooves respectively deepened palatales or only thickened lateral palatine ridges: putting the visual assessment of the palatal configuration into perspective by means of metric assessments revealed that, although oral intubation may lead to palatal grooving, palatal grooving was not necessarily associated with an increase in palatal depth [32], whereas in another study the subjective assessment of palatal height correlated reasonably well with the palatal index [59].

The above mentioned shortcomings affected the comparability of the non-metrical data and gave rise to vastly varying data on the incidence of grooving (7–90%). Subjective assessments have not the kind of discriminatory power which is nowadays desirable for identifying potential genetic, environmental or developmental associations of deformities. However, a visual inspection of the infant palate may give the pediatrician some important diagnostic clues with respect to syndromes and changed functional patterns. This is why intraoral examinations should be an integral part of postnatal pediatric routine examinations and why non-metric diagnoses were included in this review.

Fourthly, only twelve metrical studies concerning PT infants palates were found, with the methodological quality varying widely [12,15,22,32,48,49,60-65]. One had the exactness of different measuring methods as the primary interest of outcome [64], three examined the effect of protective appliances [48,49,61], four included preschool or school children of a wide age range [12,22,60,65] (one study showed the mean difference in palatal width from 9 – 12 years in girls was 0.9 mm in the molar region [66]), one measured palatal depths intraorally, entailing the risk of being imprecise [15], one study included term and preterm infants [32]. In the majority of studies a problem with the reliabilty of the measuring method was present: Either the reliabilty was not given [15,22,32,62,63], or a significant measuring error for palatal depth was recorded [6], or the coefficent of variation for repeated palatal height measurements ran up to 11.73% [49,64].

Fifthly, the confounding of prematurity, i.e. birtweight and gestational age vs intubation in most cases cannot be resolved, as many preterm infants need the latter.

Sixthly, there is the risk of at least 4 × 2 included papers being 'double publications' reffering to the same group of infants ([67] and [68]; [43] and [44]; [69] and [70]; [62] and [63]). This entails the risk of bias and impact on the conclusions of the review [71].

Seventhly, the following problems are worth to be mentioned:

• The calcium phosphate metabolism has so far been taken into account in only one study [62], the type of milk intake in only one other [49]. As two-thirds of the newborn's stores of calcium and phosphorus are accumulated during the third trimester of pregnancy, and a premature infant born prior to about 28 or 30 weeks gestation would have missed much of his mineral accretion [72], it cannot be exluded that bias came over the metrical studies on PT infants palates due to missing data on nutrition.

• The development of the palate is linked to that of the mandible and can thus not be seen in isolation [73]: the dimensions for the maxillary gum pad do vary considerably beginning with an overjet, i.e. a sagittal distance of upper and lower jaw of >6 *mm *[74].

• The development of the maxilla is linked to that of the cranial base [73] and cranium. Only two of the metric studies in PT infants [32,49] took this mutual relationship into account.

• The development of the palate is subject to various functions: in comparison with the closed mouth of the term-born infant, the mouth of the PT infant is commonly open [75], which might have a significant implication for orofacial development and was not considered in any of the studies. Attention to the influence of oral feeding was made in only one study [64].

## Conclusion

Considering the shortcomings of the currently available articles on palatal development (lack of uniform definitions of palatal morphology, lack of control studies with term infants, lack of studies with determination of the reliability of the measuring method), the following conclusions may be carefully drawn:

### The palate of the term newborn

1. The distinctive feature of the infantile palate is the groove system. The lack of uniformity in the nomenclature of the groove system and of the frenula of the infant jaw underlines the need for a uniform definition in the anatomic terminology.

2. The shape of the palate of the term infant can considerably vary, both, visually and metrically.

3. Contradictory information is given with regard to gender differences in palatal shape of term infants.

4. With the exception of one study, in which indian and latino children were included, all studies reported more palatal cysts in term white children compared to black babies. Alveolar notches and alveolar lymphangioma occur more often in black neonates. In term infants, no gender differences were found with respect to alveolar notches, palatal cysts, alveolar cysts or lymphangioma.

5. Contradictory statements were given for term infants with respect to a correlation of birthweight or gender and palatal size at birth.

6. No significant differences between spontaneously and forceps delivered term infants have been described with respect to palatal size.

7. Contradictory statements were given concerning a correlation between nasal deformity and palatal symmetry, thus no conclusions concerning that subject can be drawn in this review.

### The palate of the preterm/low birthweight infant

1. Orotracheal intubation has been reported to be harmful for teeth, tooth eruption, palatal shape and speech as early as 12 hours after intubation.

2. Due to a non-uniform definition and a subjective, non-metric evaluation in the majority of the studies there is a marked difference in the percentage data on the incidence of palatal grooving in PT infants (7 – 90%).

3. The following facts have been accused for provoking grooving: head flattening, pressure of an oral tube, pathologic or impeded tongue function and broadening of the alveolar ridges adjacent to the tube. Thickened palatine ridges may give a false impression of palatal height.

4. Metrically, the palates of intubated PT babies remain narrower, what has been examined up to the age of 11 years. Thus, an earlier orthodontic control of formerly orally intubated PT infants compared to non-intubated infants is advisable. From the orthodontic point of view, nasal intubation should be favoured.

5. Contradictory information is given in the literature on PT infants concerning

• the correlation of length of intubation time and amount of grooving.

• the duration of 'grooving' (which was examined up to the age of ten years).

• the incidence of crossbites compared to non-intubated babies.

• a possible difference in palatal asymmetry compared to non-intubated babies.

• palatal depth compared to non-intubated babies.

Thus, no conclusions are possible concerning those subjects on the base of this review.

6. It remained unclear, if gestation or birthweight of preterm infants were related to palatal height, due to confounding with intubation time.

7. Palatal plates have proven to protect palates with inserted tubes from deepening. Pressure dispersing pads for the head, however, did not have a significant impact on palatal height. It remained unclear, if changes in palatal width occurred due to pressure dispersing pads or due to oral feeding. There is a need of prospective studies to assess the infection rate and development of the tooth buds in children with protective plates.

8. PT children seem to have significantly less palatal cysts than term babies (be aware of different examination times for PT and term children and of the difficulty of an oral examination in a tiny infant!).

9. Up to the corrected age of 13.8 weeks, the palatal morphology of non-orally intubated PT infants does not differ from that of (probably) term infants. Beyond that time, no controlled long term studies comparing palatal dimensions of non-intubated PT children with those of non intubated term children are available. Thus, it cannot be excluded, that (e.g. as a consequence of functional impairment) PT infants do have a priori an altered palatal shape, which has been wrongly attributed to oral intubation.

10. No statement can be made on the development of biometric palatal data of term infants in the period from 1930 to present on the basis of the reviewed studies, as age groups were formed over several non-comparable months and data on the body height and weight of the probands were unfortunately lacking in most studies.

11. Further investigations in which the parents are also examined are needed to clarify the implication of genetic factors in the palatal configuration.

12. Parameters such as diet (breast milk versus PT formula), mode of feeding (bottle- versus breast-versus orogastric vs. nasogastric feeding), positioning, habits as well as biometric data and the influence of the mandible must be included more consistently in future studies than they have been to date.

13. Future studies should quote the product of palatal height and width in order to give a numerical expression of relative palatal height. As two palates with apparently different shapes may have an identical palatal index, the palatal length should also be included for a better three-dimensional understanding of palatal shape, too.

## List of abbreviations

[PT] preterm infant, [BW] birthweight, [LBW] low birthweight, [NBW] normal birthweight, [VLBW] very low birthweight, [NBW] normal birthweight, [GA] gestational age, [GW] gestational weeks, [NS] not significant

## Competing interests

The author(s) declare that they have no competing interests.

## Authors' contributions

AH designed the study, searched the databases, extracted the data, analyzed the results and wrote the manuscript. HR helped with study design, analysis and provided critical input in neonatal associated issues and revised the manuscript. UE and EH formulated the research question, helped with study design, analysis and in revising the manuscript. All authors read and approved the final manuscript.
